# Star-Shaped Crosslinker for Multifunctional Shape Memory Polyurethane

**DOI:** 10.3390/polym12040740

**Published:** 2020-03-26

**Authors:** Xiuhuan Song, Hong Chi, Zibiao Li, Tianduo Li, FuKe Wang

**Affiliations:** 1 Shandong Provincial Key Laboratory of Molecular Engineering, School of Chemistry and Pharmaceutical Engineering, Qilu University of Technology (Shandong Academy of Sciences), Jinan 250353, China; 2 Institute of Materials Research and Engineering, A*STAR (Agency for Science, Technology and Research), Fusionopolis Way, Innovis, #08-03, Singapore 138634, Singapore

**Keywords:** polyurethanes, shape memory, crosslinker, cyclophosphazene, flame retardance

## Abstract

Star-shaped cyclophosphazene (ACP) was employed as covalent crosslinker to form a rigid segment in polyurethanes network, to enhance the mechanical performance and to add extra flame retardant property. The effects of different ACP contents on the shape memory ability and fire resistance performance of polyurethane (PU) were studied. Tensile tests suggested high flexibility of the PUs with the maximum elongation-at-break of 161.59%. Dynamic mechanical analysis (DMA) indicated good shape recovery ratio of 72.58% after more than three repeated cycles. Under thermal treatment, the temporary shape could recover to its original state in 10 s. The peak heat release rate (pHRR), total heat released (THR) and temperature at pHRR (T_p_) of flame-retardant shape memory polyurethane (FSPU) by micro-combustion calorimeter (MCC) was as low as 183.2 W/g, 21.4 KJ/g, 330.8 °C respectively, suggesting good inherent fire-resistant performance. As amine-containing crosslinkers are one of the most common building units in thermosetting polymers, we anticipate that our finding will have significant benefits beyond shape memory and fire-resistance.

## 1. Introduction

Shape memory polymers (SMPs) are one of the most widely studied smart materials that can temporarily adopt programmed shapes and recover their original shapes upon external stimulation. The past decade has witnessed remarkable progress in SMPs with potential applications in aerospace [1], civil engineering [2], electronic engineering [3], energy [4], textiles [5], household products [5] and high-value-added engineering applications [6]. As a multifunctional polymer, shape memory polyurethanes (PUs) have attracted great attention in the scientific community and industry due to its biocompatibility and excellent mechanical properties [7,8]. Polyurethanes have a unique microphase inhomogeneity in its polymer chain with inherent soft and hard segments—the polyester-based soft segments (SS) and diisocyanate-based hard segments (HS) [9]. The hydrogen bonds of urethane groups formed in-between amino group and isocyanate group were stronger than that formed from hydroxyl group and isocyanate group, resulting in uneven microphase [10]. The HS can form a force center that holds the soft segments, retaining the original shape and playing a major role in the shape recovery process, whereas the SS retains and holds a temporary shape after the programming process [11]. When the content of HS is low, the SS is a filling matrix among HS. This results in a higher shape fixation of the SMPs. On the contrary, the HS acts as the filling matrix for the SS and the HS domains form rigid network, resulting in the lower shape fixity due to elastic property of HS [12]. Polyester, polyether or polyols was employed as chain extenders and impact on the distribution and crosslinking of soft and hard segments, resulting in different physical properties.

The presence of two different segments (hard segment and soft segment) can be influenced by the external trigger to form shape memory effect [13]. Most of polyurethanes employ physical cross-linking to fix the structure to memorize the original shape. However, the main limitation of physically cross-linked shape-memory polymers is irreversible deformation during memory programming [14]. To achieve various needs for enhancement of the shape memory functions, usage of covalent crosslinkers such as hyperbranched polyols, trimethylol propane, poly(ester imide) or other super-molecules have been proposed [15,16,17]. In this present work, we introduced the star-shaped cyclotriphosphazene with six peripheral aniline groups as crosslinker to enhance the mechanical performance and add extra flame retardant property to the resulting shape memory polyurethane. 

The cyclophosphazene derivatives composed of an inorganic phosphazene ring and various substituted organic functional groups, forming a unique cyclic structure [18]. They exhibit the properties of both inorganic and organic substances and are crucial fine chemical intermediates [19]. In addition to the anti-oxidation, reduction and biocompatibility property, cyclophosphazene derivatives also show the advantage of flame-retardance because of the halogen-free and high phosphorus-nitrogen structure [20]. This specific structure and chemical property can be used to reasonably prepare star-shape polymer with desired properties, making it widely explored in the fields of tissue engineering and flame-retardance [21]. Furthermore, cyclic triphosphazene derivatives with different substituents allow us to easily obtain multifunctional crosslinker to enhance the shape memory property of the resulting PU. It has been reported that chemical crosslinking of polymers leads to SMPs with robust shape memory, high thermal stability and chemical stability [22,23]. Here we successfully synthesized amine-terminated cyclophosphazene (ACP) by nucleophilic substitution on the phosphorus atom. By modulating the feeding ratio of ACP, a series of flame-retardant shape memory polyurethanes (FSPUs) were synthesized with ACP as a crosslinking agent. Tensile test showed enhancement in elongation and toughness with 5 wt.% loading of ACP crosslinker. Dynamic mechanical analysis (DMA) indicated good shape recovery rate of 72.58%. The microscale combustion calorimeter (MCC) suggested that the incorporation of ACP could alter the thermal degradation behavior and enhanced the flame retardancy of PU.

## 2. Materials and Methods

### 2.1. Materials

Hexachlorocyclotriphosphazene (HCCP) (98%), anhydrous potassium carbonate (K_2_CO_3_), 4-acetamidophenol (99.5%), hexamethylene diisocyanate (HDI), tris(2-aminoethyl) amine (TEAE) (96%), polypropylene glycol (PPG) (Mw = 400) were purchased from Shanghai Aladdin Biochemical Technology Co., Ltd., Shanghai, China. Sodium hydroxide (NaOH), *N*,*N*-dimethylformamide (DMF), acetone, absolute ethanol, *n*-hexane and methanol (MeOH) were purchased from Sinopharm Chemical Reagent Co., Ltd., Shanghai, China. The acetone was distilled to remove the reducing substance by adding potassium permanganate. Then the residual water was further distilled off by adding anhydrous potassium carbonate.

### 2.2. Synthesis of Hexa(acetamidophenyl) Cyclotriphosphazene (CPAC)

The procedure and schematic of the prepared structure was shown in Figure S1. A mixture of hexachlorocyclotriphosphazene (HCCP) [N_3_P_3_Cl_6_] (0.347 g, 1 mmol), 4-acetamidophenol (1.088 g, 7.2 mmol) and K_2_CO_3_ (1.492 g, 10.8 mmol) in acetone (60 mL) was heated under reflux for 72 h. The volatile reagents were removed by rotary evaporation and the residue was washed with water (3 × 20 mL), ethanol (2 × 5 mL) and hexane (3 × 5 mL), respectively. The resulted white solid powder was dried in vacuum oven at 40 °C for 48 h. Yield: 0.9 g (87%). ^1^H-NMR (DMSO-*d*6, TMS, ppm): a: 9.92 (1H, –NH), b–c: 6.79–7.45 (4H, dd, Ar–H), d: 2.04 (3H, –CH_3_) (Figure S2). MS (MALDI-TOF MS) *m*/*z* (%):1036.352 [M + H]^+^ (Figure S3).

### 2.3. Synthesis of Amine-Terminated Cyclophosphazene (ACP)

To a solution of CPAC (0.518 g, 0.5 mmol) in methanol (20 mL) was added a solution of NaOH (2.4 g) in water (3 mL). The mixture was heated under reflux for 24 h. The resulting white powder was filtered and washed with a large excess of water, ethanol (3 × 5 mL) and hexane (3 × 5 mL), respectively before dried in vacuum oven at 40 °C for 48 h. Yield: 0.27 g (70%). The ^1^H-NMR of the amine-terminated cyclophosphazene (ACP) is as follows: (DMSO-*d*6, TMS, ppm): a: 4.91 (2H, Ar–NH_2_), b–c: 6.41–6.52 (4H, dd, Ar–H) (Figure S4). MS (MALDI-TOF MS): 784.240 [M + H]^+^ (Figure S5). 

### 2.4. Preparation of Polyurethane Films with and without ACP

**Figure 1:**
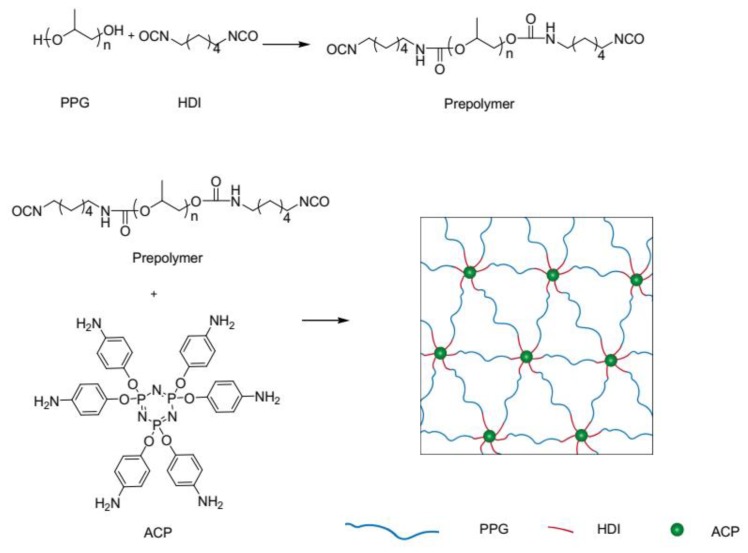
Schematic illustration of the preparation procedure and the designed fire-retardant shape memory polyurethane network.

As shown in Figure 1, PPG was selected because of excellent chain mobility and solubility [23]. The polymer synthesized by using tris(2-aminoethyl) amine as crosslinker exhibited better thermal stability and mechanical adjustability [24], so it was employed in synthesizing neat PU for comparison in this work. In a typical experiment for the preparation of FSPU with 5 wt.% ACP (FSPU-5), a mixture of HDI and PPG was heated to 65 °C in nitrogen atmosphere and lasted for 55 min to prepare the prepolymer of PU. Then 5 wt.% ACP dissolved in 5 mL DMF was added. The mixture was magnetically stirred for 30 min before cast into a PTFE mold. After that, the reactants were allowed to be cross-linked at 60 °C for 48 h. Then, a flat cross-linked FSPU film could be obtained as the final product. Neat PU films were prepared in same manner by using tris(2-aminoethyl) amine as crosslinker. With this method, a laboratory made film with diameter as bigger as 15 cm could be easily obtained and the FSPUs showed good transparency in visible light range (Figure S6). Table 1 showed the feeding ratio of each reactant in preparation of a series of FSPUs as well as neat PU.

### 2.5. Methods

IR spectroscopy: Fourier-transform infrared spectra (FT-IR) were recorded on a Perkin Elmer 2000 Fourier transform infrared (FTIR) spectrometer (Bruker, Karlsruhe, Germany) with a frequency range of 4000–500 cm^−1^.

Nuclear magnetic resonance: Proton nuclear magnetic resonance (^1^H-NMR) spectra were recorded in DMSO using a Bruker AMX-400 spectrometer (Bruker, Karlsruhe, Germany) at a frequency of 400 MHz. 

High resolution mass spectrometry (HRMS): High resolution mass spectrometry (HRMS) were recorded using ultraflextreme™ MALDI-TOF/TOF (ultrafleXtreme MALDI-TOF/TOF, Bruker, Karlsruhe, Germany) mass spectrum with dithranol-NaI as matrix compound.

Thermogravimetric analysis (TGA): Thermogravimetric analysis (TGA) of the PU films was conducted on SDT-Q600 (TA Instruments, New Castle, DE, USA) from 40 °C to 600 °C at a heating rate of 10 °C/min in N_2_ atmosphere. 

Tensile tests: The spline of the PU film is stretched at a force of 5 N per minute under the room temperature by a computer controlled electronic universal testing machine (Hensgrand, WDW-02, Jinan, China).

Dynamic mechanical analysis (DMA): DMA measurements were conducted in a tensile mode using DMA Q800 from TA Instruments (TA Instruments, New Castle, DE, USA). A frequency of 1 Hz was used for all measurements. The samples were loaded on a clamp and measured under nitrogen at a heating rate of 5 °C/min from −70 °C to 120 °C. The storage modulus E′ and loss factor tan δ was tested with specimen size 30 mm (length) × 5 mm (width) × 0.3 mm (thickness).

Shape memory test: The shape memory properties were evaluated with a DMA Q800 dynamic mechanic analyzer (TA Instruments, New Castle, DE, USA). In free strain recovery experiments, the tested specimen was clamped with an initial length of 30 mm in-between the grips of the tensile testing machine. Before starting, the compression stress was reduced to zero by moving one clamp until the specimen was in its unloaded state (initial strain *ε*_p_ = 0). Then the specimen was heated to T_high_ = 70 °C using a heating rate of 5 °C·min^−1^ (±0.6 °C·min^−1^) and held for 5 min before the shape programming process was started. After that, the specimen was elongated to an absolute strain *ε*_m_ of 100%, using a strain rate of 0.050 S^−1^ (±0.002 S^−1^). Strain changes during heating process were included in *ε*_m_. After holding the specimen at that temperature for 5 min, the constrained specimen was cooled to T_low_ = −10 °C (±1 °C) with a cooling rate of 5 °C·min^−1^ (±0.6 °C·min^−1^) and kept under constant strain for another 5 min. Unloading the specimen with a rate of 5.0 N·min^−1^ (±0.4 N·min^−1^) and holding for 5 min to complete the shape programming process. Measure the strain *ε*_u_ of the new unconstrained temporary shape. Shape recovery was initiated by reheating the specimen to 70 °C with same heating rate. The onset temperature of shape (free strain) recovery was defined by the value of the tangent line through the inversion point of the shape recovery curve at the foregoing temporary force *ε*_u_ in the strain-time diagram. The corresponding temperature was taken as onset shape (free strain) recovery temperature T_r,onset_. The residual strain associated with the recovered permanent force *ε*_p_ was measured after 5 min at T_high_. The entire first thermo-mechanical cycle (N = 1) was finished after this step. Subsequently, another 3 cycles were run with same elongation of the specimen. 

Pyrolysis combustion flow calorimetry (PCFC): PCFC texts were performed using a FAA Microscale combustion calorimeter (MCC) (Fire Testing Technology, East Grinstead, UK) according to ASTM D7390.5 ± 0.5. The samples were heated from 100 to 700 °C at a heating rate of 1 °C/s at a nitrogen flowing rate of 80 mL/min. 

## 3. Results and Discussions

### 3.1. Structural Characterization

**Figure 2:**
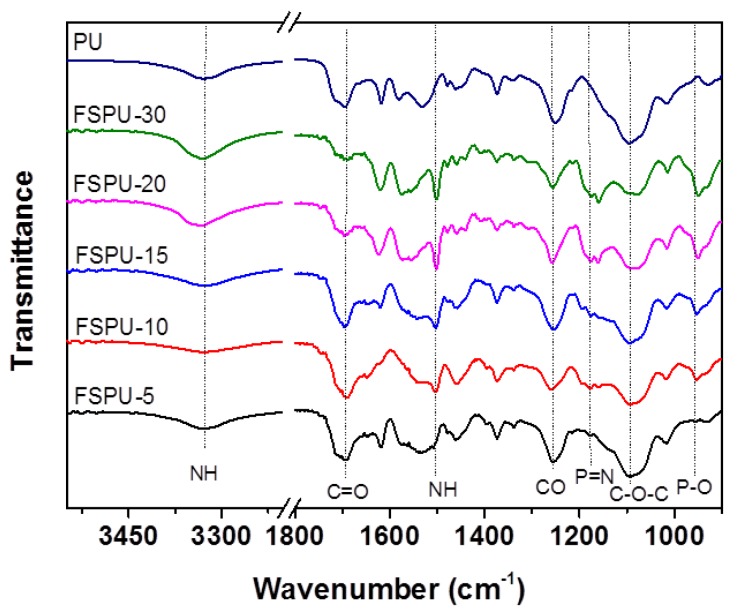
Fourier-transform infrared spectra (FT-IR) spectra of the neat and star-shaped cyclophosphazene (ACP) incorporated PU films.

The infrared spectra were collected to interpret the polymerization reaction of FSPUs. As shown in Figure 2, no characteristic stretching vibration peak of P=N and P–O at 1177 cm^−1^ and 955 cm^−1^ was found in the neat PU. As the feeding ratio of ACP increases in the FSPUs, the transmittance of P=N and P–O peaks increased from FSPU-5 to FSPU-30, indicating more ACP was incorporated into the matrix of PU. Other characteristic peaks of polyurethane were also found. The stretching vibration of C=O, C–O and O–C–O in the ester group appeared at 1710 cm^−1^, 1250 cm^−1^ and 1120 cm^−1^, respectively.N–H vibration was found at 3300 cm^−1^. The vibration peak at 1540 cm^−1^ was assigned to the newly formed urea bond.

### 3.2. Thermal Behavior Analysis

**Figure 3:**
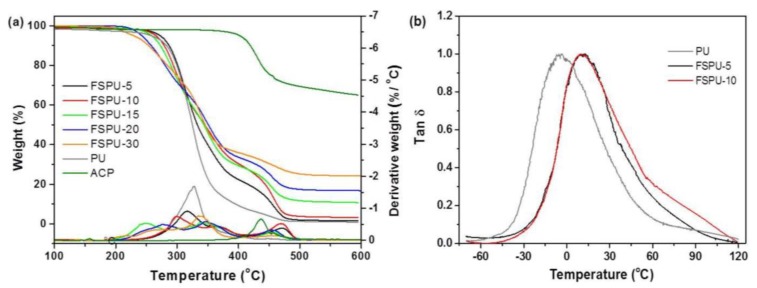
(**a**) Thermogravimetry/Derivative thermogravimetry (TG/DTG curves of neat PU and FSPUs with various ACP contents and (**b**) Tan *δ* curves of neat PU, FSPU-5 and FSPU-10.

To probe the thermal degradation profiles and figure out how they are affected by ACP, TGA was run and the Thermogravimetry/Derivative thermogravimetry (TG/DTG) curve was shown in Figure 3a. Two distinguishable reaction stages of neat PU were confirmed by the presence the two combustion process where the maximal decomposition rates located at 327 °C and 449 °C, respectively. Such two-stage degradation agrees well with literature [25,26] that indicated that the first stage is the decomposition of polymer chains and the release of volatile compounds, while the second stage represents the oxidation process of the residues. Only one weight loss stage in ACP was found at 437 °C. As shown in the DTG curve, the intensity of peaks that correspond to the decomposition rate was obviously affected by the ratio of ACP. The peaks move to higher temperature as the ACP content increases, indicating that the addition of ACP reduced the interaction between free chains which may be assigned to the cross-linking of the prepolymer. Therefore, the decomposition of both free prepolymer and polymer matrix was slower than neat PU. The second peak moved from 449 °C up to 470 °C which is higher than either ACP or neat PU, suggesting synergistic effect existed between PU and ACP. It was speculated that the free prepolymer decomposed first, followed by the segments on ACP and terminated when the fragmentation on the skeleton of P–O–C decomposed [27]. 

As summarized in Table S1, increased decomposition temperature (T_onset_) was found in FSPU-5 and FSPU-10 but started to decrease from FSPU-15. This change might due to weakened inter-chain interactions [28]. To investigate how the star-shaped cross-linker affects the polymer performance, we fixed the feeding ratio of the PPG to HDI with 1:2 for the purpose to get the prepolymer with ending isocyanate groups. Prepolymers with ending isocyanate groups were reported to exhibit good coating property [9,29], also they were used to provide active groups to crosslink with ACP (one aniline group can react with two isocyanate groups) here. No other crosslinkers were added to FSPUs to simplify the study. As compared with ACP, the isocyanate groups are excess in FSPU-5, FSPU-10 and FSPU-15. This is to ensure that all ACP can be cross-linked into the polymer network and also a higher degree of cross-linking can be reached. Therefore, the rigidity of FSPUs would increase because of higher crosslinking density. This would possibly restrict the urethane bond interaction, resulting lower thermal stability of the urethane group as well as the initial decomposition temperature. The remaining residuals agreed well with the calculated feeding ratio, suggesting that all ACP was reacted. The glass transition temperatures (T_g_) related to the soft segment structure in the tested PUs were obtained from the tan *δ* peak. Due to the brittle nature of FSPU-15, FSPU-20 and FSPU-30, no free-standing films of these samples were obtained and used for DMA tests. As shown in Figure 3b, all tan *δ* curves displayed one peak, indicating similar amorphous structure in these three cross-linked PUs. The neat PU showed lower T_g_ than FSPU-5 and FSPU-10, indicating easier motion of chain segment crosslinked by TEAE. FSPU-5 and FSPU-10 exhibited nearly the same T_g_ at 11.3 °C, which may be possibly due to close chain length in the prepolymer and stronger restriction by ACP. According to the crosslinking density ν_e_ and the storage modulus E of the rubber plateau region, the following relationship exists: ν_e_ = E/(6RT), measured at T_g_ + 40 °C [30]. It can be deduced that ν_eFSPU-5_/ν_eFSPU-10_ = 0.11, so the crosslinking density increased. E_FSPU-5_ = 16.09 Mpa, ν_eFSPU-5_ = 994.29 mol/m^3^; E_FSPU-10_ = 145.32 Mpa, ν_eFSPU-10_ = 9027.45 mol/m^3^, where ν_e_ is the crosslinking density in mol/m^3^; E is the storage modulus of the rubber plateau region above T_g_, the unit is 0.1 Pa; R is the gas constant, 8.314 J·mol^−1^·K^−1^; T is the absolute temperature in K. With the addition of ACP, the crosslink density increased from FSPU-5 to FSPU-10, thus a wider peak in tan *δ* was observed. The T_g_ of FSPUs is higher than neat PU, because that the increased steric hindrance in FSPUs limited the molecular chain rotation.

### 3.3. Mechanical Performance Tests

#### 3.3.1. Tensile Test

**Figure 4:**
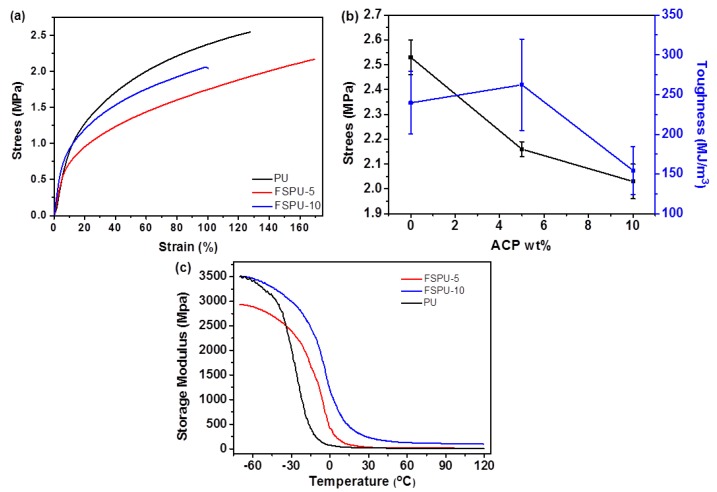
Tensile properties of neat PU and FSPUs with various ACP contents: (**a**) stress-strain curve, (**b**) stress and toughness versus ACP addition curve and, (**c**) E’ curves of neat PU, FSPU5 and FSPU-10.

FSPUs exhibited significant elastic behavior due to their high soft segment ratio (Figure 4a,b). As summarized in Table 2, the tensile strength and elongation of FSPUs are significantly affected by the ratio of ACP. As the content of ACP increases, larger rigidity and smaller elongation at break were observed which was attributed to the increased crosslinking between the prepolymers. Therefore, FSPU with more than 10 wt.% ACP exhibited premature failure in tensile tests, even though the amount of ACP was lesser than the corresponding stoichiometric ratio in samples containing up to 15% ACP (Table 1). Therefore, samples with higher feeding of ACP could not be tested. An optimized toughening effect was found in FSPU-5 with higher flexibility and moderate tensile strength. 

#### 3.3.2. Shape Memory Cycles

It is well known that the mechanical of thermosetting polymers is sensitive to the chemical structure and nature of copolymerized systems [31]. DMA was used to study the organization of chain segments and relationship between structure and mechanical performance. Variation of the storage modulus (E’) as the function of temperature was shown in Figure 4c. At low temperatures, the PUs exhibited a glassy character. The molecular segmental motions were frozen and E’ remained high. With temperature increasing, the frozen segmental structure began to relax gradually and all E’ values of the PUs were tending to balance at above 50 °C. FSPU-10 showed the highest E’ because of more crosslinker. FSPU-5 with less crosslinker and more free prepolymer showed lower rigidity and exhibited better flexibility in tensile tests.

**Figure 5:**
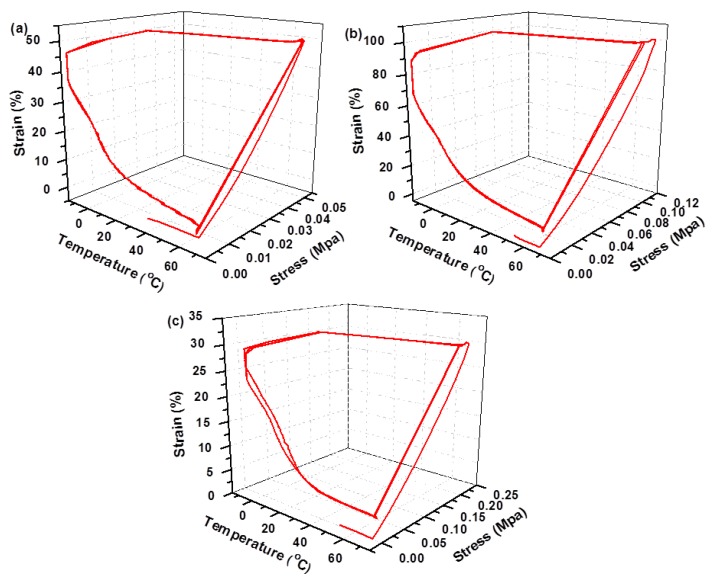
Shape memory cycle of (**a**,**b**) FSPU-5 and (**c**) FSPU-10. Shape memory cycles of FSPUs were performed where each sample was stretched at 70 °C and fixed at −10 °C, followed by recovering at 70 °C in a stress-controlled mode via dynamic mechanical analysis (DMA).

All the selected samples exhibited large variations in modulus with temperature changes, which is essential requirement for shape memory applications by benefiting from the deformation at high temperature and fixing temporary shape at low temperature. To assess the performance of the FSPUs as shape memory materials, DMA measurement of FSPU-5 and FSPU-10 were conducted in a controlled force mode. We chose 70 °C as the recovery temperature because most thermoset shape memory polymers can achieve perfect recovery at this temperature [32]. To fix the shape and get same Tan *δ*, we found a symmetrical point which is −13.2 °C from Tan *δ* curve. Therefore, −10 °C was employed experimentally as fix temperature. As plotted in Figure 5, FSPUs were initially heated to T_trans_ = 70 °C. At this temperature, the entanglement of PPG molecular chains was removed and the polymers were easy to be deformed. Then the samples were subjected to certain strains with different stress (step i). To thermally fix this strain, the samples were cooled down to −10 °C under constant stress (step ii). At −10 °C, the stress was released (step iii) and the samples retained a considerable portion of the strain. Upon heating back to 70 °C, the strain was released and the sample was recovered to the original shape, exhibiting certain degree of recovery (step iv).

**Figure 6:**
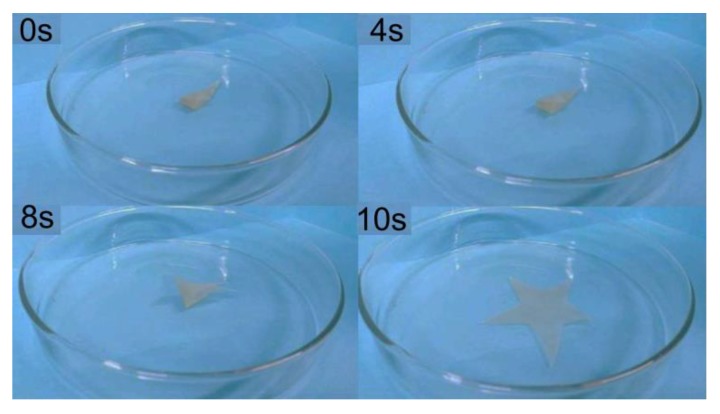
Visual demonstration of the shape recovery process of the FSPU-5 under heating at 60 °C.

The incomplete recovery of both samples is because the orientation of chain-segment was irreversible and the relaxation effect in the polymer network was partly dissipated by the stored entropy [33,34]. Compared with some thermosetting shape memory materials with recovery rates of 40–49% and 42% in literature [35,36], FSPU-5 exhibited excellent shape fixity and the shape recovery ratio (72.58%) after three cycles. This is possibly due to lower crosslinking density and more prepolymers existing between crosslinking points to store residual strain. The irregular arrangement of hard segments at higher strains was no longer sufficient for effective recovery. Therefore, large deformation forces may cause partial plastic deformation of the segments, which cannot be recovered at 70 °C. The images in Figure 6 visually demonstrated the shape recovery process of FSPU-5. 

As indicated in Figure 6, the sample almost can finish a complete cycle at 60 °C. To demonstrate the shape memory effect, we wish to use lower heating temperature and high fixing temperature experimentally. Therefore, 60 °C and 0 °C were employed, respectively. At the beginning, the star shape sample was heated to 60 °C before being deformed into a curved shape. Immediately thereafter, the deformed sample was placed in an ice water mixture (0 °C) for about 600 s to fix the temporary shape. After bringing back to the heat source, they return to the original shape. The FSPU-5 slowly opened from the initial fixed state and recovered within 10 s. The video S1 in the support information also showed the shape deformation and recovery process.

### 3.4. Flame Retardant Evaluation

To assess the performance of the FSPUs as flame retardant materials, microscale combustion calorimeter (MCC) based on oxygen consumption calorimetry was conducted by pyrolysis combustion flow calorimetry (PCFC) [37]. MCC can quickly and easily provide the key flammability parameters of materials, such as peak heat release rate (pHRR), total heat released (THR) and temperature at pHRR (T_p_) [37,38]. The MCC data of PUs with different contents of ACP were presented in Table 3. It can be concluded that the addition of ACP reduced the pHRR and THR values compared to neat PU obviously. The pHRR values of the FSPU samples were reduced from 393.4 W/g of neat PU to 183.2 W/g, respectively with increasing amount of ACP incorporated. Compared with neat PU, the THR value of FSPUs also decreased from 35.9 KJ/g to 21.4 KJ/g, which was reduced by 40.38%. The T_p_ of FSPUs decreases only when higher amount of ACP existed. This is possibly because the addition of ACP promoted the early decomposition of PU and formed a protective carbon layer [39]. The MCC results are basically consistent with the TGA analysis.

## 4. Conclusions

In summary, star-shaped cyclotriphosphazene with aniline groups (ACP) were synthesized and used as a cross-linking agent and reactive flame retardant in a new series of shape memory polyurethanes. Compared with neat PU that cross-linked by triamine, the addition of ACP ranging from 5 wt.% to 10 wt.% could enhance both tensile strength and thermal stability. Further increase of ACP induced over crosslinking and stiffened the polymer matrix. DMA tests indicated good shape recovery rate of FSPU-5 with 72.58% after more than three repeated cycles. Microscale combustion calorimeter (MCC) indicated the increase of residual carbon residue with the increase of ACP contents, suggesting an ideal combination of the flame retardant and shape memory properties into PU materials.

## Figures and Tables

**Table 1:** Feeding ratio of raw materials in the preparation of neat polyurethane (PU) and flame-retardant shape memory polyurethanes (FSPUs).

Polyurethane	PPG-400 (mmol)	HDI (mmol)	ACP (mmol)	ACP (%)	Feeding Ratio	Stoichiometric Ratio
PU	8.3	16.5	0 (2.2 *)	0	1:2:0	1:2:0
FSPU-5	8.3	16.5	0.41	5	1:2:0.05	1:2:0.167
FSPU-10	8.3	16.5	0.83	10	1:2:0.10	1:2:0.167
FSPU-15	8.3	16.5	1.24	15	1:2:0.15	1:2:0.167
FSPU-20	8.3	16.5	1.63	20	1:2:0.20	1:2:0.167
FSPU-30	8.3	16.5	2.45	30	1:2:0.30	1:2:0.167

Note: * Amount of TEAE triamine crosslinker in neat PU.

**Table 2:** Tensile data of the FSPUs with different content of ACP.

Sample	Tensile Strength (MPa)	Elongation at Break (%)	Toughness (MJ/m^3^)
PU	2.53 ± 0.43	120.34 ± 12.8	239.58 ± 39.55
FSPU-5	2.16 ± 0.03	161.51 ± 9.29	262.28 ± 57.47
FSPU-10	2.03 ± 0.07	102.34 ± 10.7	154.43 ± 30.04

**Table 3:** Microscale combustion calorimeter (MCC) results of PUs with different contents of ACP.

Sample	Peak HR (W/g)	Total HR (KJ/g)	T_p_ (°C)
PU	393.4	35.9	368.9
FSPU-5	408.6	26.2	369.0
FSPU-10	361.8	23.8	371.2
FSPU-15	212.0	23.1	371.1
FSPU-20	193.9	21.5	361.7
FSPU-30	183.2	21.4	330.8

## Supplementary Materials

The following are available online at https://www.mdpi.com/2073-4360/12/4/740/s1, Figure S1: Schematic illustration of the synthesis procedure for ACP, Figure S2: 1H-Nuclear magnetic spectrum of hexa(acetamidophenyl) cyclotriphosphazene (CPAC), Figure S3: MALDI-TOF/TOF mass spectrum of hexa(acetamidophenyl) cyclotriphosphazene (CPAC), Figure S4: 1H-Nuclear magnetic spectrum of amine-terminated cyclophosphazene (ACP), Figure S5: MALDI-TOF/TOF mass spectrum of amine-terminated cyclophosphazene (ACP), Figure S6: Transmittance spectra of FSPU-5 and FSPU-10 in visible light range. The inset image indicated the large size and transparency of FSPU-5 made in the laboratory by film casting method, Table S1: Thermal properties of neat PU and FSPUs with various ACP contents, Video S1: Shape memory demo.
