# Supplementary material for: Star-Shaped Crosslinker for Multifunctional Shape Memory Polyurethane
Source: Polymers (Basel). 2020 Mar 26;12(4):740. doi: 10.3390/polym12040740 (PMC7240373; doi:10.3390/polym12040740)
Supplement: Supplementary file 1 [file polymers-12-00740-s001.zip › Supplementary information/Revised Supplementary Information.doc]

Supplementary Information

**Star-shaped Crosslinker for Multifunctional Shape Memory Polyurethane**

Xiuhuan Song ^1^, Hong Chi ^1,*^, Zibiao Li ^2^, Tianduo Li ^1^ and Fuke Wang^2,*^

*1	Shandong Provincial Key Laboratory of Molecular Engineering, School of Chemistry and Pharmaceutical Engineering, Qilu University of Technology (Shandong Academy of Sciences), Jinan, China 250353*

*2	Institute of Materials Research and Engineering, A*STAR (Agency for Science, Technology and Research), 2 Fusionopolis Way, Innovis, #08-03, Singapore 138634, Singapore*

**Figure S1.** Schematic illustration of the synthesis procedure for ACP.

**Figure S2.** 1H-Nuclear magnetic spectrum of hexa(acetamidophenyl) cyclotriphosphazene (CPAC).

**Figure S3.** MALDI-TOF/TOF mass spectrum of hexa(acetamidophenyl) cyclotriphosphazene (CPAC).

**Figure S4.** 1H-Nuclear magnetic spectrum of amine-terminated cyclophosphazene (ACP).

**Figure S5.** MALDI-TOF/TOF mass spectrum of amine-terminated cyclophosphazene (ACP).

**Figure S6.** Transmittance spectra of FSPU-5 and FSPU-10 in visible light range. The inset image indicated the large size and transparency of FSPU-5 made in the laboratory by film casting method.

**Table S1. Thermal properties of neat PU and FSPUs with various ACP contents.**

| **Polyurethane** | **T_oneset_ (^o^C)** | **Residue at 600 ^o^C**  **(wt%)** | **T_g_ (^o^C)** |
| --- | --- | --- | --- |
| PU | 264.4 | 0.75 | -3.8 |
| ACP | 402.2 | 65.04 | -- |
| FSPU-5 | 272.9 | 1.49 | 11.4 |
| FSPU-10 | 265.6 | 3.17 | 9.7 |
| FSPU-15 | 258.5 | 10.73 | -- |
| FSPU-20 | 242.3 | 16.73 | -- |
| FSPU-30 | 227.1 | 24.13 | -- |

T_onset_: Initial decomposition temperature (based on 5 wt% loss)
